# Identification of Key Transcription Factors AP-1 and AP-1-Dependent miRNAs Forming a Co-Regulatory Network Controlling PTEN in Liver Ischemia/Reperfusion Injury

**DOI:** 10.1155/2019/8962682

**Published:** 2019-11-05

**Authors:** Yiming Zhong, Youwei Zhu, Junfeng Dong, Wei Zhou, Cheng Xiong, Meilin Xue, Minmin Shi, Hao Chen

**Affiliations:** ^1^Department of General Surgery, Ruijin Hospital, Shanghai Jiao Tong University School of Medicine, Shanghai, China; ^2^Research Institute of Digestive Surgery, Ruijin Hospital, School of Medicine, Shanghai Jiao Tong University, Shanghai, China; ^3^Department of Organ Transplantation, Changzheng Hospital, Second Military Medical University, Shanghai, China; ^4^Department of Biochemistry and Molecular Cell Biology, Shanghai Key Laboratory of Tumor Microenvironment and Inflammation, Shanghai Jiao Tong University School of Medicine, Shanghai, China

## Abstract

Liver ischemia/reperfusion (I/R) injury is a complex and common clinical disease with limited therapeutic options. The aim of our study was to discover the candidate target genes in liver I/R injury and to further elucidate the potential regulatory mechanisms, especially the ones involving transcription factors and miRNAs. The analysis of mouse data set GSE10657 from Gene Expression Omnibus database (GEO) revealed 203 differentially expressed genes (DEGs) including 19 transcription factors (TFs). Functional and pathway enrichment analyses were conducted to explore their biological functions. We further obtained the targets of TFs and miRNAs, to form our TF-mRNA/TF-miRNA-mRNA co-regulatory network. In our network, we found that the important subunits of activator protein 1 (AP-1) including JUN, FOS and ATF3, were hub genes in liver I/R injury. AP-1 target genes were activated in our mouse models. AP-1 could transcriptionally activate phosphatase and tensin homolog (PTEN) while AP-1-dependent miRNAs countered this effect. In conclusion, this study suggested that AP-1, together with AP-1-dependent miRNAs formed a co-regulatory network enabling AP-1 target genes to be tightly controlled, which will complete the mechanism of liver ischemia/reperfusion injury and provide direction for finding potential therapeutic targets.

## 1. Introduction

Liver surgery is often accompanied by hepatic ischemia/reperfusion, and liver ischemia/reperfusion injury is a common pathological process which results in liver dysfunction in the early stages after transplantation [1, 2]. However, the specific mechanism of the occurrence and development of liver ischemia/reperfusion is still largely unclear.

The popularity of genome-wide sequencing, the promotion of chip technology, and the continuous progress of bioinformatic analyses in the recent years enable us to construct the regulatory network of miRNA and TFs. It provides us a new approach to uncover the regulatory mechanisms of liver I/R injury [3]. JUN, an important subunit of AP-1 [4], is reported as a key gene in the process of liver ischemia/reperfusion [5, 6]. Moreover, we identified PTEN as a target gene of AP-1 in our own regulation network. PTEN is well known to counter-regulate phosphoinositide 3-kinase (PI3K) activity, which is crucial in cell survival and growth [7]. PTEN inhibition has been reported to increase I/R survival and reduce injury [8]. Our study validated one of the potential regulatory mechanisms suggested by our bioinformatic analyses, the AP-1/PTEN and AP-1/miRNAs/PTEN co-regulatory network in mouse models. We found that AP-1, together with AP-1-dependent miRNAs formed a co-regulatory network enabling tight control of AP-1 target genes. Therefore, the core TFs along with their regulatory network suggested in our regulatory network may become potential targets for future liver ischemia/reperfusion therapy.

## 2. Materials and Methods

### 2.1. Microarray Data

The expression microarray data of GSE10657 [9] were obtained from Gene Expression Omnibus (GEO), which had been background adjusted and normalized. GSE10657 included 15-mice (4-5 weeks), and mice were divided into five groups, respectively: Sham, Ischemia 30 min (Ish_30 min), Ischemia 60 min (Ish_60 min), Ischemia 90 min (Ish_90 min), and Ischemia 90 min + reperfusion 60 min (Ish_90 min_rpf). In order to observe the whole dynamic process of hepatic ischemia/reperfusion, we paired different treatment groups in the dataset according to the biological process of ischemia/reperfusion for differential expression analysis (Ish_30 min vs. Sham, Ish_60 min vs. Ish_30 min, Ish_90 min vs. Ish_60 min, Ish_90 min_rpf vs. Ish_90 min, Ish_90 min_rpf vs. Sham).

### 2.2. Differential Expression Analysis

The processing of probe-level data in CEL files was conducted by the affy package in *R* [10]. Background correction was carried out using the robust multi-array average (RMA) method, followed by quantile normalization and probe summarization [11]. The limma package was applied to identify differentially expressed genes (DEGs) between two groups. In this analysis, adjusted *p* value <0.05 and |log2 (fold change, FC)|>1 were used as the cutoff criteria. Transcriptional factors were selected from DEGs according to the Tfcheckpoint database[12].

### 2.3. GO & KEGG Enrichment

To study the pathway and biological process of these 203 genes, the metascape webserver [13] was hired to perform the functional annotation, Gene Ontology (GO) enrichment analysis and Kyoto Encyclopedia of Genes and Genomes (KEGG) pathway enrichment analysis. The threshold of significant differences was set at an adjusted *p* value ≤0.01 after Benjamini correction. The heatmap *R* package was employed to exhibit the significant enrichment terms. The top 10 enriched items (sorted by Generatio, a proportion of enriched gene count to total DEGs) in GO-BP, GO-CC, GO-MF, and KEGG pathway were visualized, respectively.

### 2.4. Construction of AP-1-Subunit-Centered Regulatory Network

In order to obtain AP-1 target genes, targets of JUN, FOS, and ATF3 were extracted from HARM-ENCODE project (http://amp.pharm.mssm.edu/Harmonizome/), respectively. To collect genes involved in ischemia/reperfusion injury from AP-1 target genes, we gathered candidate genes from two sources including identified DEGs from our differential analysis and results from an extensive literature search. PUBMED search using keywords “(ischemia/reperfusion injury) AND genes” revealed 498 genes. Then we searched “ischemia/reperfusion injury” in DisGeNet database (http://www.disgenet.org/home/) to generate gene set associated with ischemia/reperfusion injury. Overlapping genes of pubmed and DisGeNet were considered as genes highly related to pathology of ischemia/reperfusion injury and used as one of the sources to construct our AP-1-subunit-centered regulatory network ([Supplementary-material supplementary-material-1]). Then we predicted the targets of miRNAs that are AP-1 target genes using TargetScan (release 7.2: March 2018) [14]. After deciphering TF-mRNA/miRNA and miRNA–mRNA regulatory relations, AP-1-subunit-centered regulatory network was constructed. Cytoscape (version 3.4.0) was used to visualize our regulation network. CentiScaPe app [15], a plug-in of Cytoscape, was used to calculate the degree distribution of network.

### 2.5. Liver I/R Modeling and GPT Assay

Male C57/BL6 mice (6 weeks old) were purchased from the Chinese Academy of Sciences. All experimental animals did not carry special pathogenic factors, and the experimental animal ethics were emphasized throughout the experiment. During the entire operation of liver ischemia/reperfusion, we adopted a nonlethal method of blocking the branches of the hepatic portal vein as previously described [16, 17], each group contained 6 mice. Group of sham animals underwent the following operations: anesthesia, laparotomy, and exposure. GPT assay was carried out using the ALT/GPT kit (nuctech, China) according to standard protocol.

### 2.6. Western Blotting Assay

Liver tissue from mice subjected to liver ischemia/reperfusion was homogenized by a homogenizer, and the protein was extracted by RIPA buffer mixed with phenylmethylsulfonyl fluoride (NCM Biotech, China) and protease inhibitor cocktail (Sigma, USA). Western blotting assay was strictly conducted in accordance with standard protocol. The protein concentration of each sample was determined by BCA Kit (Pierce, USA). The 10% SDS-PAGE gel was used to separate the protein and PVDF (Tanon, China) was used to transfer the protein. Those nonspecific binding sites were sealed by skim milk. Blots were probed with Anti-PTEN (No. 9188, Cell Signaling Technology, USA), Anti-p-c-JUN (No. 3270, Cell Signaling Technology, USA), Anti-GAPDH (No. 5174, Cell Signaling Technology, USA).

### 2.7. RNA Isolation and Quantitative Real-Time PCR

The entire RNA extraction and quantitative real-time PCR process was strictly performed according to the previous study [18]. Briefly, total RNA was extracted from liver tissue using TRIzol (Invitrogen, USA). The quality and concentration of total RNA was detected by the spectrophotometer (Bio-Rad, USA). The cDNA was then synthesized using the kit of ReverTra Ace qPCR RT (TOYOBO, Japan). Finally, quantitative real-time PCR was performed using SYBR Green (TOYOBO, Japan). *β*-ACTIN was used as an internal reference. Each gene was tested for at least three independent experiments. The primer sequences we used were as follows: PTEN: (forward primer) 5'-TGGATTCGACTTAGACTTGACC-3' and (reverse primer) 5'-TCACTTAGCCATTGGTCAAGAT-3', c-JUN: (forward primer) 5'-GGGAGCATTTGGAGAGTCCC-3' and (reverse primer) 5'-TTTGCAAAAGTTCGCTCCCG-3', ATF3: (forward primer) 5'-GTCACCAAGTCTGAGGCGG-3' and (reverse primer) 5'-GTTTCGACACTTGGCAGCAG-3', MMP9: (forward primer) 5'-GGAGCACGGCAACGGAGAAG-3' and (reverse primer) 5'-CCTGGTCATAGTTGGCTGTGGTG-3'.

In order to isolate the miRNA, we used the miRNA Extractor (QIAGEN, Germany) to obtain miRNA from the liver tissue. The quality and concentration was also detected by the spectrophotometer (Bio-Rad, USA). Kit of miScript II RT (QIAGEN, Germany) was used to synthesize cDNA according to the instruction. The detection of miRNA expression was carried out using miRNA Quantitation PCR Kit (QIAGEN, Germany). Endogenous control of miRNA was U6. The primers for target miRNAs were purchased from Sangon Biotech (China).

### 2.8. Cell Culture, Hypoxic Reoxygenation Modeling and Reagents

Mouse hepatic parenchymal cell AML12 was purchased from ATCC. AML12 was cultured in DMEM/F12 with 10% fetal bovine serum (FBS; Gibco, USA) and 1% ITS (Insulin, Transferrin, Selenium, Cyagen, USA). AML12 cell were maintained at 37°C under stable 5% CO_2_ in a humidified chamber.

For hypoxia and reoxygenation modeling, AML12 was cultured in hypoxic incubator (94% N 2.5% CO_2_ and 1% O_2_) for 90 min and then stimulated with 100 ng/ml LPS (Sigma-Aldrich, USA) and 20 *μ*M H_2_O_2_ in normal incubator.

### 2.9. Plasmid Constructs and Luciferase Reporter Assay

pAP1-TA-luc reporter gene plasmid was purchased from Beyotime Biotechnology (China). PTEN promoter-luc reporter gene plasmid and Luc-PTEN 3'UTR reporter gene plasmid were synthesized by Genechem (China). Luciferase activities in different treatment groups were measured through the Dual-Luciferase Reporter Assay System (Promega, USA) 48 h after transfection according to the manufacturer's instructions. Renilla luciferase served as an internal reference.

### 2.10. Statistics

Data were expressed as mean ± standard error of mean (SEM). Statistical significance between two groups was determined by unpaired *t*-tests and between multiple groups was determined by ANOVA, (^∗^*P* < 0.05, ^∗∗^*P* < 0.01, ^∗∗∗^*P* < 0.001 and ^∗∗∗∗^*P* < 0.0001). Data analyses mainly used SPSS13.0 (SPSS Inc. Chicago, USA) and GraphPad Prism 6.0 software (GraphPad, Inc., San Diego, CA, USA).

## 3. Results

### 3.1. Differentially Expressed Genes (DEGs) in Liver I/R Based on GEO Database

**Figure 1:**
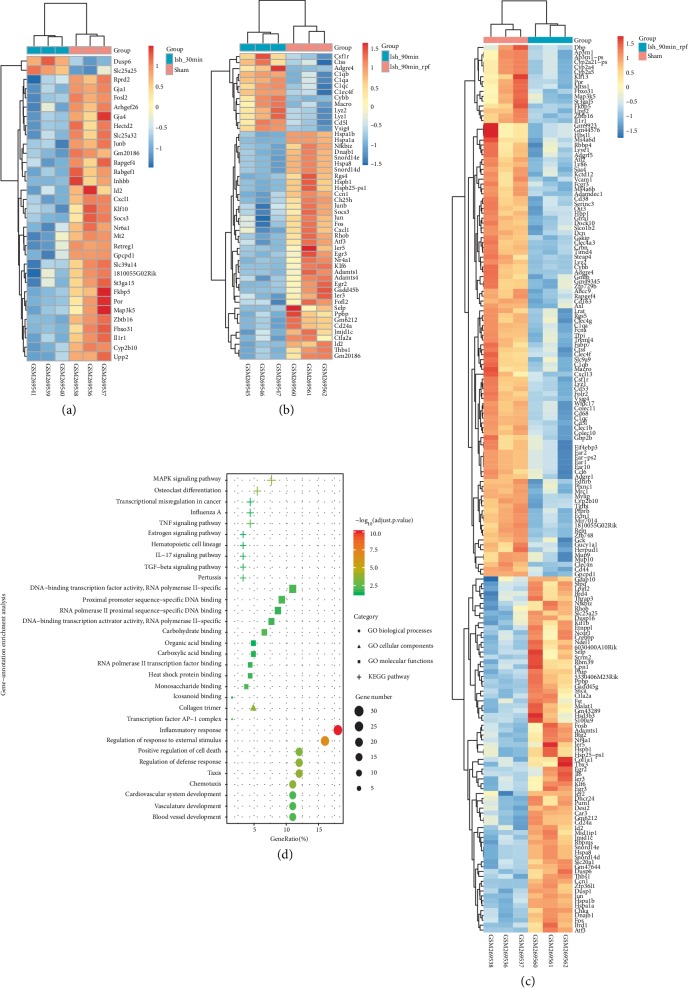
Identified DEGs in GSE10657 dataset and gene function enrichment analysis between different comparisons. (a) Hierarchical clustering heatmap of DEGs between Ish_30 min vs. Sham. (b) Heatmap of Ish_90 min_rpf vs. Ish_90 min. (c) Heatmap of Ish_90 min_rpf vs. Sham. Orange represents that the expression of genes is relatively up-regulated and blue represents that the expression of genes is relatively down-regulated. (d) The top 10 enrichment scores in KEGG pathway and gene ontology (GO) enrichment analysis. The horizontal axis shows the gene-ratio of the selected genes and the vertical axis represents the enriched terms. The bigger the plots are, the more genes are enriched in this term.

Series matrix files of GSE10657 were obtained from GEO. According to the biological process of ischemia/reperfusion injury, we identified 33 DEGs in Ish_30 min vs. Sham, of which 2 were up-regulated, 31 were down-regulated (Figure 1(a) and [Supplementary-material supplementary-material-1]), and there was no DEG in Ish_60 min vs. Ish_30 min or in Ish_90 min vs. Ish_60 min. Differential analysis of Ish_90 min and Ish_90 min_rpf revealed 51 DEGs, of which 38 were up-regulated, 13 were down-regulated (Figure 1(b) and [Supplementary-material supplementary-material-1]). Finally, among Ish_90 min_rpf and sham, we confirmed 182 DEGs, including 73 up-regulated genes and 108 down-regulated genes (Figure 1(c) and [Supplementary-material supplementary-material-1]). The DEGs of these groups were combined together. A total of 203 differential genes were obtained, 82 were up-regulated and 127 were down-regulated, of which 6 genes (Socs3 Fosl2 Junb Gm20186 Id2 Cxcl1) were down-regulated during the ischemic phase and up-regulated during the reperfusion phase. Notably, among these 203 differentially expressed genes (DEGs), there are 19 TFs (Table 1).

### 3.2. Functional Analysis of the DEGs

After identifying the DEGs, we conducted functional enrichment analysis of these 203 DEGs (Figure 1(d)). As shown in Figure 1(d), KEGG pathway analysis revealed that mitogen-activated protein kinase (MAPK) signaling pathway was a distinctly enriched pathway. Moreover, GO annotation showed DEGs were significantly enriched in biological process of inflammatory response, regulation of response to external stimulus, and positive regulation of cell death; cellular components of GO showed that DEGs were enriched in collagen trimer and transcription factor AP-1 complex (Figure 1(d)).

### 3.3. AP-1-Subunit-Centered Regulatory Network

**Figure 2:**
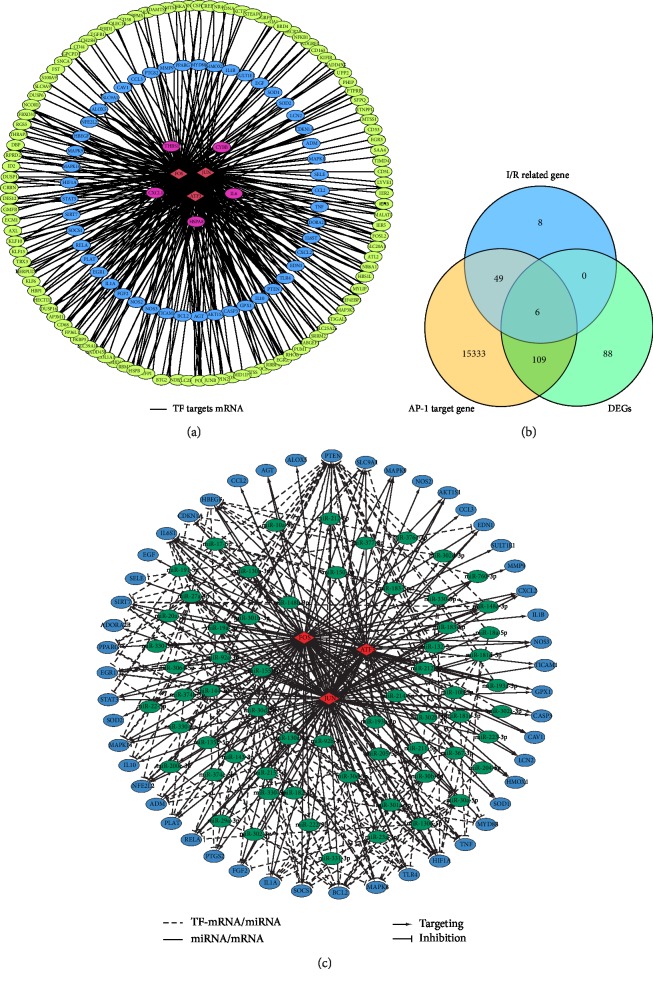
AP-1-subunit-centered regulatory network. (a) Representation of AP-1-subunit-centered regulatory network. Yellow circles: differentially expressed genes (DEGs). Blue circles: ischemia/reperfusion (I/R) injury related genes. Purple circles: genes that are both DEGs and I/R injury related genes. Red diamonds: transcription factors (TFs). (b) Venn diagram representation of overlap of I/R related genes, AP-1 target genes and DEGs. (c) AP-1-mRNA/miRNA and miRNA–mRNA regulatory network. Blue circles: mRNAs. Green circles: miRNAs. Red diamonds: TFs.

As we all know, hub nodes play critical roles in biological networks. Our bioinformatic analyses identified JUN and FOS, which are subunits of AP-1, as differentially expressed TFs after liver ischemia/reperfusion injury. JUN and FOS are targets of MAPK pathway [19]. Furthermore, KEGG pathway revealed MAPK signaling pathway as one of the most significantly enriched pathways (Figure 1(d)), suggesting that AP-1 played an important regulatory role in the process of liver I/R. Based on these results, we decided to construct the AP-1-subunit-centered regulatory network. We incorporated experimentally verified AP-1 target genes from HARM-ENCODE project. Considering that liver I/R is a dynamic and acute process, DEGs retrieved from samples of set time point may not fully represent this complex biological process and may omit genes of important biological functions. To overcome this issue, in addition to DEGs from our analysis, we gathered candidate genes from an extensive literature search to construct the regulatory network (Figure 2(a) and [Supplementary-material supplementary-material-1]).

### 3.4. AP-1 and AP-1 Target Genes Were Activated during the Liver I/R Injury

**Figure 3:**
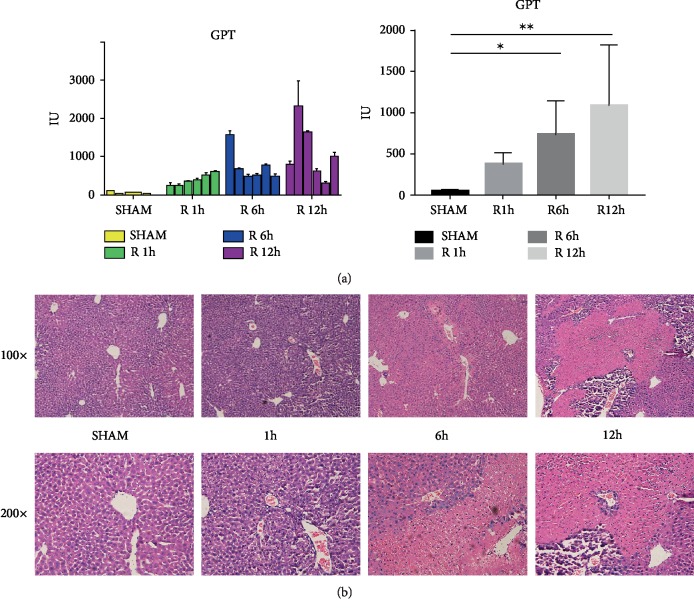
Mice liver ischemia/reperfusion (I/R) induce liver injury. (a) GPT levels of mice liver I/R in sham group, reperfusion 1 h, reperfusion 6 h, reperfusion 12 h. (b) Hematoxylin and eosin stain (HE) analysis of mice liver. ^∗^*P* < 0.05, ^∗∗^*P* < 0.01, ^∗∗∗^*P* < 0.001.

**Figure 4:**
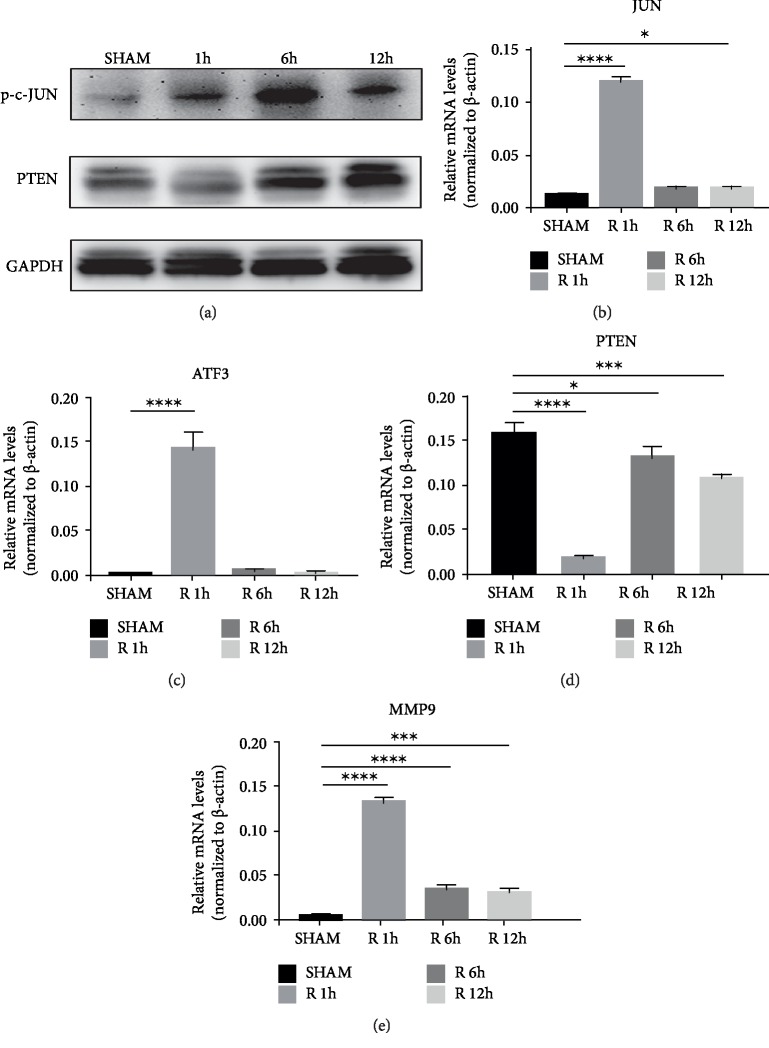
Activation of AP-1 and AP-1 target genes in the process of liver ischemia/reperfusion (I/R) injury. (a) PTEN, p-c-JUN, GAPDH expression were examined by a western blot assay. (b, c) mRNA levels of AP-1 subunits (JUN and ATF3). (d, e) AP-1 target genes, including PTEN, MMP9. ^∗^*P*< 0.05, ^∗∗^*P* < 0.01, ^∗∗∗^*P* < 0.001 and ^∗∗∗∗^*P* < 0.0001.

We next performed a nonlethal segmental ischemia/reperfusion model on the mice, and the different treatment groups were given ischemia for 90 min, reperfusion for 1 hour, 6 hours, and 12 hours. Serum glutamate pyruvate transaminase (GPT) levels were significantly elevated 6 hours after modeling (Figure 3(a)). Histological damage and inflammatory response in liver tissue could be observed about 6 h after reperfusion by hematoxylin and eosin (HE) staining (Figure 3(b)). Our heatmap of Ish_90 min_rpf vs. Ish_90 min and Ish_90 min_rpf vs. Sham showed that JUN, an important subunit of AP-1, was one of the hub genes in our regulation network of the liver I/R injury. Activation of AP-1 requires phosphorylation of AP-1 subunits [4]. Phosphorylation of c-JUN was detected after reperfusion (Figure 4(a)), which suggested that AP-1 was activated at the early stage of mice liver I/R injury. Consistent with our heatmap (Figures 1(b) and 1(c)), mRNA levels of AP-1 subunits (JUN and ATF3) were evidently elevated in I/R groups (Figures 4(b) and 4(c)).

In order to better verify the results of our network, we performed qPCR to detect the mRNA levels of several AP-1 target genes that have been reported to have biological significance in I/R injury, including MMP9 and PTEN [20–22]. As predicted by our bioinformatic network, MMP9 was significantly up-regulated in our I/R model (Figure 4(e)). However, one of the AP-1 target genes, PTEN were down-regulated in the early stage of liver I/R injury (Figure 4(d)). Although PTEN was predicted as a target gene of AP-1 and considered as one of genes highly related to pathology of ischemia/reperfusion injury by our literature search, our differential analysis failed to identify PTEN as a DEG (Figure 2(b)). We hypothesized that in addition to being the target gene of AP-1, PTEN is co-regulated by miRNAs to account for this phenomenon. Therefore, we predicted the targets of miRNAs that are transcriptionally activated by AP-1 using TargetScan (release 7.2: March 2018). We focused on the 49 candidate genes that our differential analysis failed to identify as DEGs like PTEN (Figure 2(b)) and reconstructed our network by adding miRNA–mRNA regulatory relations (Figure 2(c) and [Supplementary-material supplementary-material-1]). The degree distribution of network was calculated to evaluate the importance of a gene in the regulatory network. PTEN has a degree of 31 while MMP9 has a degree of 1 in our regulatory network ([Supplementary-material supplementary-material-1]) suggesting PTEN was likely to be regulated in a much more complex manner.

### 3.5. PTEN Was Transcriptionally Activated by AP-1 in Cellular Hypoxia/Reoxygenation Model

**Figure 5:**
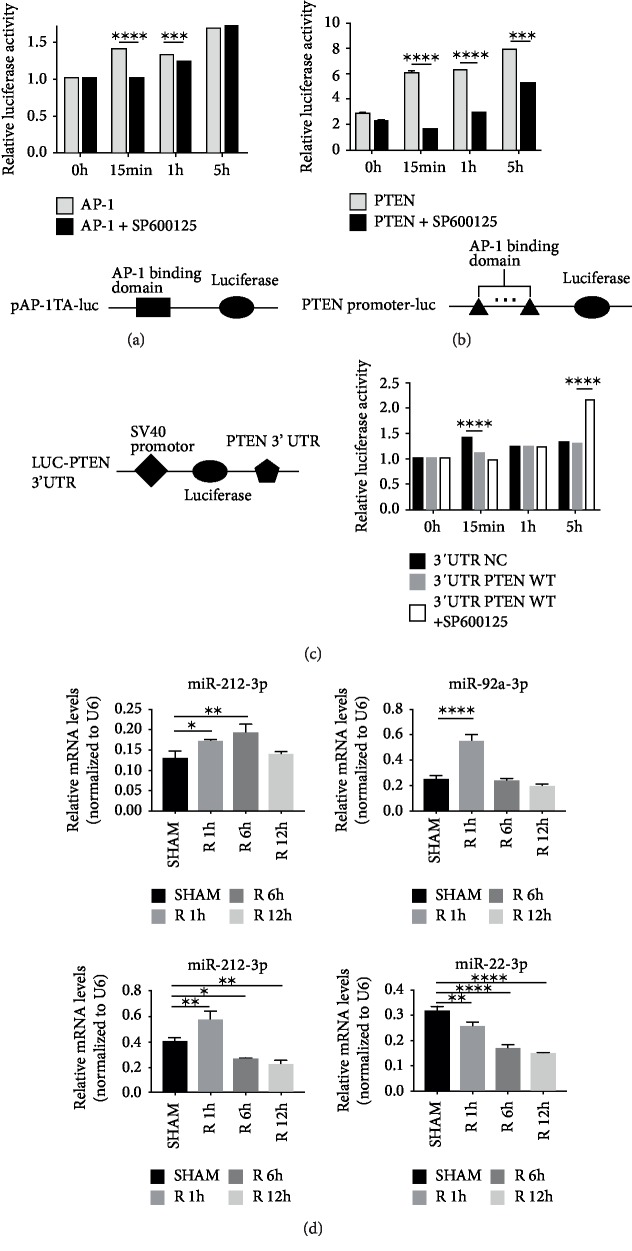
PTEN was transcriptionally activated by AP-1 while inhibited by AP-1-dependent miRNAs in cellular hypoxia/reoxygenation model. (a) Results of AP-1 reporter gene luciferase assay. (b) Results of PTEN promoter luciferase reporter assay. (c) Results of the luciferase reporter assay of 3'UTR of PTEN. Cellular hypoxia/reoxygenation model was used in luciferase reporter assays. (d) miR-212-3p, miR-22-3p, miR-29a-3p, miR-92a-3p expression levels in mice liver I/R sample were examined by qPCR. SP600125 is a Jun N-terminal kinase (JNK) inhibitor. ^∗^*P* < 0.05, ^∗∗^*P* < 0.01, ^∗∗∗^*P* < 0.001, ^∗∗∗∗^*P* < 0.0001.

To investigate the regulatory mechanism of PTEN in liver I/R injury, we simulated the physiological process of liver I/R injury in vitro by employing a cellular hypoxia/reoxygenation model in which AML12 cells were cultured in a hypoxic incubator for 90 min and next stimulated with LPS and H2O2 for 15 min, 1 h and 5 h in the normal oxygen environment. Luciferase reporter assay revealed that AP-1 was activated as early as 15 minutes. The activation of AP-1 was hindered by Jun N-terminal kinase (JNK) inhibitor SP600125 (Figure 5(a)). We constructed PTEN promoter luciferase reporter plasmid containing 7 putative JUN binding sites (Table 2), and PTEN promoter luciferase reporter assay showed that PTEN could be transcriptionally activated during cellular hypoxia/reoxygenation. This activation was significantly inhibited by SP600125 (Figure 5(b)). Considering AP-1 is a major target of JNK signaling pathway [4], these findings suggested that PTEN was transcriptionally activated by AP-1 in hypoxia/reoxygenation model in vitro.

### 3.6. AP-1-Dependent miRNAs Target 3'UTR of PTEN in Cellular Hypoxia/Reoxygenation Model

To investigate whether miRNA is involved in the regulation of AP-1/PTEN axis, we constructed the specific luciferase reporter gene plasmid containing 3'UTR of PTEN. To best mimic the regulation of PTEN, we employed SV40 promoter, which can also be activated by AP-1, in our luciferase reporter gene plasmid. As expected, we observed elevated luciferase activity in the control group after hypoxia/reoxygenation modeling, while luciferase activity in PTEN 3'UTR group was significantly decreased at 15 min after reoxygenation compared with control group (Figure 5(c)). The decrease of luciferase activity in PTEN 3'UTR group was significantly reversed at 5 h after reoxygenation when treated with JNK inhibitor (Figure 5(c)). Taken together, these lines evidence suggested that AP-1-dependent miRNAs targeted 3'UTR of PTEN in vitro.

**Figure 6:**
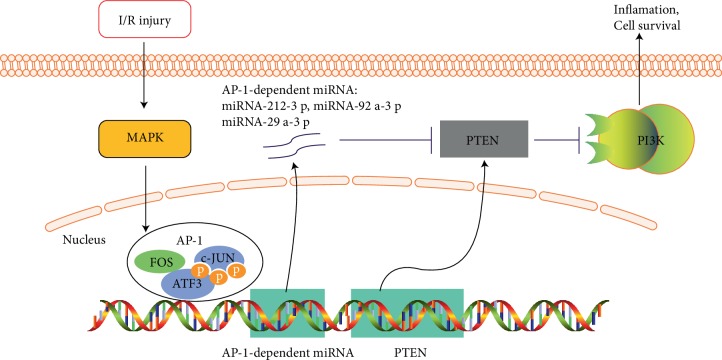
Model of AP-1/ AP-1-dependent miRNAs/PTEN regulation mechanism. In the hepatic ischemia/reperfusion, AP-1 is activated, which activates downstream PTEN. At the same time, AP-1-dependent miRNAs are also activated. These miRNAs have targeted PTEN and negatively regulate PTEN. In this way, AP-1 forms a very precise regulation of PTEN during hepatic ischemia/reperfusion.

According to the results obtained above, we speculated that AP-1-dependent miRNAs can target 3'UTR of PTEN during I/R injury. To test our hypothesis in vivo, we selected several AP-1-dependent miRNAs that were predicted to target PTEN (miR-212-3p, miR-92a-3p, miR-29a-3p, and miR-22-3p), which were also predicted to be the target genes of AP-1 in our network. Next, we verified their expression levels in liver I/R tissues by qPCR. The expression levels of miR-212-3p, miR-92a-3p, miR-29a-3p, and miR-22-3p were markedly altered in the early stage of liver I/R injury (Figure 5(d)). These results indicated that AP-1-dependent miRNAs that were predicted to target PTEN were altered in liver I/R injury and possibly played an important role in regulating PTEN expression at the post-transcriptional level (Figure 6).

## 4. Discussion

Our study aimed to construct the interaction network of miRNA and TF in mice liver I/R model, which could provide new targets and regulatory mechanisms for further experiment and clinical therapy. When we used high-throughput data to find the hub genes in biological processes, the expression of some key genes often fluctuate so that they cannot be captured at specific sampling points. Therefore, identifying key genes only by high-throughput data may lack biological significance [23]. The direct connection between nodes and nodes could be clearly demonstrated in the regulatory network. Intuitive concepts of regulation network, such as module and connectivity, have been used to analyze the complicated interactions. As a result, the above concepts and methods had been applied in many fields, for example, analyses of gene co-expression networks [24], protein-protein interaction networks [25, 26], and cell-cell interaction networks [27]. In our current study, we focused on differentially expressed TFs and identified their targets to form our TF-mRNA/miRNA and miRNA–mRNA co-regulatory network.

Based on network theory, TFs and miRNAs often function in interconnected pathways at multiple levels rather than function in isolation. Here we validated our hub TFs in mouse models and found that important subunits of AP-1 (JUN, FOS and ATF3), were crucial TFs in the regulation network of liver I/R. In addition, we found that PTEN, a target gene of AP-1, could be regulated by AP-1 at multiple levels. PTEN could be transcriptionally activated by AP-1 and negatively regulated by AP-1-dependent miRNAs at the post-transcriptional level during cellar hypoxia/reoxygenation modeling. This co-regulatory network centered by AP-1 could explain why PTEN was de-regulated in the early stage of I/R injury, unlike many other AP-1 target genes. PTEN can aggravate liver I/R injury through restraining AKT pathway [28, 29]. Early inhibition of PTEN may play a protective role via up-regulating PI3K/Akt pathway in liver I/R injury. The above results suggested that AP-1-dependent miRNAs may act a significant role in down-regulating PTEN at early stage of liver I/R injury.

Many studies had previously reported that miRNAs could participate in the process of ischemia/reperfusion by regulating the expression of PTEN. miR-21 had been reported to target PTEN in liver and cardiac I/R [30]. Su et al. [31] reported that miR-494 could attenuate liver I/R injury by targeting PTEN and up-regulating PI3K/Akt pathway in rat I/R injury. miR-182-5p alleviated liver I/R via suppressing TLR4 [32]. However, the activation mechanism of these miRNAs remained unclear [33]. Our miRNA–mRNA regulatory network revealed that AP-1-depandent miRNAs were also involved in AP-1/PTEN regulatory axis, allowing tight regulation of PTEN. Further study is required to confirm whether other AP-1 target genes are regulated in the same manner.

There are some limitations in our study. We showed that AP-1-dependent miRNAs were involved in AP-1/PTEN pathway, however further research is required to identify the specific AP-1-dependent miRNA targeting PTEN. We suspect that multiple miRNAs may be involved in the regulation.

In summary, we identified key transcription factors in I/R injury and formed our TF-mRNA/miRNA and miRNA–mRNA co-regulatory network. During validation of our network, we found that AP-1, together with AP-1-dependent miRNAs formed a co-regulatory network enabling AP-1 target genes such as PTEN to be tightly controlled. Further study is required to confirm whether other AP-1 target genes are regulated in the same manner.

## 5. Conclusions

Collectively, our study suggested that AP-1, together with AP-1-dependent miRNAs formed a co-regulatory network enabling AP-1 target genes to be tightly controlled, which will complete the mechanism of liver ischemia/reperfusion injury and provide direction for finding potential therapeutic targets.

## Figures and Tables

**Table 1:** Identified differentially expressed TFs. (Comparison: 1: Ish_30 min vs. Sham, 2: Ish_60 min vs. Ish_30 min,3: Ish_90 min vs. Ish_60 min, 4: Ish_90 min_rpf vs. Ish_90 min, 5: Ish_90 min_rpf vs. Sham)

Gene symbol	Gene name	Comparison
EGR3	Early growth response 3	4,5
HBP1	HMG-box transcription factor 1	5
ZBTB16	Zinc finger and BTB domain containing 16	1,5
IER2	Immediate early response 2	5
CREBBP	CREB binding protein	5
ATF3	Activating transcription factor 3	4,5
NR4A1	Nuclear receptor subfamily 4, group A, member 1	4,5
JUNB	Jun B proto-oncogene	1,4
NFKBIZ	NFKB inhibitor, zeta	4,5
FOS	FBJ osteosarcoma oncogene	5
KLF6	Kruppel-like factor 6	4,5
JUN	Jun proto-oncogene	4,5
FOSB	FBJ osteosarcoma oncogene B	5
BTG2	B cell translocation gene 2, anti-proliferative	5
TBX3	T-box 3	5
FOSL2	Fos-like antigen 2	1,4
EGR2	Early growth response 2	4,5
KLF13	Kruppel-like factor 13	5
DBP	D site of albumin promoter (albumin D-box) binding protein	5

**Table 2:** PTEN promoter luciferase reporter plasmid containing 7 putative JUN (Model ID: MA0488.1) binding sites.

Binding sites	Score	Relative score	Start	End	Strand	Predicted site sequence
1	9.924	0.896	41	53	1	GAGTTGATGTCAT
2	1.799	0.807	44	56	−1	AAAATGACATCAA
3	2.213	0.812	313	325	−1	CCGATGTTGCAAC
4	1.799	0.807	392	404	−1	TTAATGAGGTGAA
5	6.175	0.855	880	892	−1	AGGAGGAGGTCAC
6	4.463	0.836	1412	1424	1	CCGATGAGGTGAC
7	3.255	0.823	1753	1765	1	GCGCTGAGGCCAA

## Data Availability

The data used to support the findings of this study are included within the article.

## Supplementary Materials

Supplementary Materials See the Supplementary Material for comprehensive image analysis.
